# Bibliometric analysis of most cited Peyronie's disease and its management publications

**DOI:** 10.3389/fsurg.2024.1336391

**Published:** 2024-05-17

**Authors:** Mehmet Fatih Şahin, Çağrı Doğan, Murat Akgül, Cenk Murat Yazıcı, Serkan Şeramet, Hulusi Sıtkı Dayısoylu

**Affiliations:** Department of Urology, Tekirdag Namık Kemal University Medical School, Tekirdağ, Türkiye

**Keywords:** Peyronie’s disease, number of citations, bibliometric analysis, adjusted citation index, altmetric score

## Abstract

**Introduction:**

Peyronie's disease (PD) is a common urologic illness, motivating numerous scientific investigations and publications. Scientific publications have more authors each year. A bibliometric review of the PD literature might help urologists and sexual medicine professionals comprehend publication tendencies in this subject. The current study was aimed at presenting a bibliometric analysis of PD, which is one of the important and trending subjects of andrology.

**Methods:**

On January 5, 2023, Web of Science scanned documents with the terms Peyronie's disease” “Peyronie's disease treatment”, “Peyronie's disease management”, “Peyronie's disease surgery” and “Peyronie's disease injection” from 1975 through 2023. Titles, years, authors, citations, citation indices, journal names, authors' countries of origin, article categories, and funding sources were recorded.

**Results:**

“Clinical Efficacy, Safety and Tolerability of Collagenase Clostridium Histolyticum for the Treatment of Peyronie Disease in 2 Large Double-Blind, Randomized, Placebo Controlled Phase 3 Studies” has the most citations and citation index. Most of the T100 articles were published in 2020, primarily in the Journal of Urology. These articles mainly focused on treatment, especially surgeries. All of these articles were in English, and the vast majority of them were by authors from the US who were most frequently collaborated with by other authors.

**Conclusion:**

This research analyzed the top 100 PD studies. This research focused on pathophysiology, innovative surgical procedures, and new approaches of PD. It also recommended bigger databases and more financing for research.

## Introduction

Peyronie's disease (PD) is an acquired condition of the tunica albuginea with painful erections, curvature, and sexual dysfunction (1). It has a prevalence rate of 0.5%–20.3%, which may differ among different populations (2). PD is believed to occur due to microtrauma of the erect penis involving a wound and a scar (3). In diagnosis, although new methods are being developed to reveal possible causes such as calcified plaques, the number of studies on this subject is still limited (4). Oral pharmacotherapy is no longer recommended for treatment. Interferon alpha 2b and collagenase clostridium histolyticum (CCH) are the preferred injection therapies for patients who do not want surgery or who prefer minimal invasive interventions. Surgical procedures such as tunical lengthening, incision or excision with grafting, and tunical shortening are also possible. The disease itself causes pronounced physical and psychological distress for patients.

Bibliometric analysis is the study of data produced about a particular topic over a specific amount of time. This type of analysis enables a more comprehensive evaluation of the current literature and improved tracking of relevant material. The bibliometric technique facilitates the identification of the most significant authors, articles, publications, nations, and organizations in a specific field of research as well as the simultaneous analysis of several papers. In their future research, authors can save time by using bibliometric analysis, as it can be employed to summarize the most pertinent literature. Additionally, bibliometric analysis can highlight current patterns of treatment and give academics fresh insights. Authors who want to contribute to the literature by examining previous studies and their details can produce more distinguished and quality works with bibliometric analyses (5). Citations are helpful for gauging the quality of studies, interpreting scientific knowledge, and discovering new and evolving subjects that can constitute the focus of future research. They also provide useful quantitative data (6). Citation analysis is a recognized technique for evaluating the significance of a research paper, researcher, journal, nation, or year (7).

Great contributions are being made to the literature with the increasing number of urology studies, especially in the field of andrology. Although classical bibliometric analyses on andrological subjects such as erectile dysfunction and PD exist, there has been not much analysis on PD, despite it being a frequently occurring condition. Accordingly, the aim of the present study was to perform a bibliometric analysis of the top 100 (T100) most cited articles on PD that were published between 1975 and 2023 in order to identify significant advancements and trends in the field and to contribute to the methodologies and hypotheses included in subsequent research.

## Materials and method

On January 5th, 2023, we searched Web of Science (Clarivate Analytics, Philadelphia, USA) papers published between 1975 and 2023 using the keywords “Peyronie's disease” “Peyronie's disease treatment”, “Peyronie's disease management”, “Peyronie's disease surgery” and “Peyronie's disease injection”. As a limitation, the study eliminated other databases like Google Scholar and Scopus, which would have had an impact on the quantity of papers returned, and we only used the Web of Science database. We have listed the resulting publications from the highest to the lowest according to the number of citations, with the most cited article residing at the top of the results list. Since the data for this study came from published literature, no ethical committee approval was necessary. The T100 articles were found among the search results for PD based on the quantity of citations. Two separate researchers (MFŞ and ÇD) independently analyzed the papers' abstracts and/or contents on any areas of disagreement to reach a consensus. Starting with the study that had the most citations, we examined each study individually. Those unrelated to PD were disregarded. We eliminated 23 records out of 123 articles as they were not relevant to the topic, making the 100th most cited article the 123rd on the list. For bibliometric analysis, the title of the article, the corresponding author, the source title, the publication year, the total number of citations, the number of articles, and the Adjusted Citation Index (ACI) were noted. In order to determine the ACI, which represents the yearly average number of citations, the number of citations was divided by the number of years since the article's publication. With the help of VOSviewer, co-authorship and co-citation analysis for the authors, co-authorship analysis for the authors according to country, and co-occurrence analysis for the authors according to keywords were analyzed. Finally, with the help of the Altmetric Bookmarklet, all of the Altmetric scores (AmS) of the publications were noted. The statistical analysis of the variables was performed by SPSS version 26.0 (IBM Corp., USA). The continuous variables of the study were summarized as medians and interquartile ranges (IQR), and the categorical variables were summarized as frequencies and percentages.

## Results

The total number of publications after the search was 3,149, all of which were published between 1975 and 2023. The most cited T100 publications are listed in Table 1. The top 100 median ± IQR citation count was 98.50 ± 52 (range: 68–287) and the median ± IQR of the ACI was 5.05 ± 3.37 (range: 2.24–26.09). The oldest publication year was 1985 and the newest was 2016. The total number of authors ranged between 1 and 43 with a median ± IQR of 4 ± 3. The median AmS was found to be 2.0 ± 4.0 (range: 0–88). After the Spearman correlation, we found that there exists a positive but relatively weak correlation between the AmS and the number of citations (Spearman's rho = 0.334, %95 CI: 0.15–0.5, *p* < 0.001, *n* = 100).

**Table 1:** The top 100 cited articles about peyronie's disease (PD).

#	PN	CA	ST	PY	TC	NoA	ACI	AmS
1	Clinical Efficacy, Safety and Tolerability of Collagenase Clostridium Histolyticum for the Treatment of Peyronie Disease in 2 Large Double-Blind, Randomized, Placebo Controlled Phase 3 Studies	Gelbard, Martin	Journal of Urology	2013	287	9	26.09	29
2	Summary of the recommendations on sexual dysfunctions in men	Lue, Tom F	Journal of Sexual Medicine	2004	276	20	13.8	3
3	The prevalence of Peyronie's disease: results of a large survey	Schwarzer, U	BJU International	2001	267	6	11.61	88
4	Subjective and objective analysis of the prevalence of Peyronie's disease in a population of men presenting for prostate cancer screening	Mulhall, JP	Journal of Urology	2004	265	8	13.25	3
5	Proposal: Trauma as the cause of the Peyronie's lesion	Devine, CJ	Journal of Urology	1997	262	4	9.7	3
6	The Management of Peyronie's Disease: Evidence-based 2010 Guidelines	Ralph, David	Journal of Sexual Medicine	2010	257	7	18.36	5
7	An analysis of the natural history of Peyronie's disease	Mulhall, JP	Journal of Urology	2006	251	3	13.94	8
8	Peyronie's Disease: AUA Guideline	Nehra, Ajay	Journal of Urology	2015	233	15	25.89	18
9	Summary of the Recommendations on Sexual Dysfunctions in Men	Montorsi, Francesco	Journal of Sexual Medicine	2010	224	43	16	4
10	Peyronie's disease is associated with an increase in transforming growth factor-beta protein expression	ElSakka, AI	Journal of Urology	1997	189	5	7	0
11	EAU Guidelines on Penile Curvature	Hatzimouratidis, Konstantinos	European Urology	2012	174	8	14.5	3
12	The Incidence of Peyronie's Disease in Rochester, Minnesota, 1950 Through 1984	Lindsay, Mb	Journal Of Urology	1991	174	6	5.27	3
13	The chronology of depression and distress in men with Peyronie's disease	Nelson, Christian J.	Journal of Sexual Medicine	2008	170	6	10.63	11
14	A retrospective review of 307 men with Peyronie's disease	Kadioglu, A	Journal of Urology	2002	164	6	7.45	0
15	Collagenase Versus Placebo in The Treatment of Peyronie's Disease—A Double-Blind-Study	Gelbard, MK	Journal of Urology	1993	153	4	4.94	6
16	A surgical algorithm for the treatment of Peyronie's disease	Levine, LA	Journal of Urology	1997	148	2	5.48	0
17	A New Treatment for Peyronie's-Disease—Modeling the Penis Over an Inflatable Penile Prosthesis	Wilson, SK	Journal of Urology	1994	147	2	4.9	3
18	The anatomy of the tunica albuginea in the normal penis and Peyronie's disease	Brock, G;	Journal of Urology	1997	145	5	5.37	3
19	Use of intralesional verapamil to dissolve Peyronie's disease plaque: A long-term single-blind study	Rehman, J	Urology	1998	141	3	5.42	6
20	A Population-Based Study of Peyronie's Disease: Prevalence and Treatment Patterns in the United States	DiBenedetti, Dana Britt	Advances In Urology	2011	140	5	10.77	0
21	Single-blind, multicenter, placebo controlled, parallel study to assess the safety and efficacy of intralesional interferon alpha-2b for minimally invasive treatment for Peyronie's disease	Hellstrom, Wayne J. G.	Journal of Urology	2006	140	13	7.78	1
22	New Surgical-Treatment for Peyronie Disease	Essed, E	Urology	1985	140	2	3.59	0
23	Impact of Peyronie's disease on sexual and psychosocial functioning: Qualitative findings in patients and controls	Rosen, Raymond	Journal of Sexual Medicine	2008	139	7	8.69	5
24	Penile trauma: An etiologic factor in Peyronie's disease and erectile dysfunction	Jarow, JP	Journal of Urology	1997	136	2	5.04	3
25	Risk factors for emotional and relationship problems in Peyronie's disease	Smith, James F.	Journal of Sexual Medicine	2008	134	5	8.38	7
26	Intralesional Verapamil Injection for The Treatment of Peyronie's-Disease	Levine, LA	Journal of Urology	1994	133	3	4.43	0
27	An animal model of Peyronie's-like condition associated with an increase of transforming growth factor beta mRNA and protein expression	ElSakka, AI	Journal of Urology	1997	129	6	4.78	6
28	Fibrin deposition in Peyronie's disease plaque	Somers, KD	Journal of Urology	1997	129	2	4.78	3
29	Venous patch graft for Peyronie's disease. Part I: Technique	Lue, TF	Journal of Urology	1998	128	2	4.92	1
30	Evidence-Based Management Guidelines on Peyronie's Disease	Chung, Eric	Journal of Sexual Medicine	2016	122	10	15.25	6
31	Peyronie's disease: A review	Gholami, SS	Journal of Urology	2003	122	5	5.81	0
32	Experience with intraplaque injection of verapamil for Peyronie's disease	Levine, LA	Journal of Urology	2002	121	3	5.5	3
33	Peyronie's disease: Etiology, medical, and surgical therapy	Hellstrom, WJG	Journal Of Andrology	2000	121	2	5.04	0
34	The Nesbit Operation for Peyronie's-Disease—16-Year Experience	Ralph, DJ	Journal of Urology	1995	121	3	4.17	0
35	Bother and Distress Associated with Peyronie's Disease: Validation of the Peyronie's Disease Questionnaire	Hellstrom, Wayne J. G.	Journal of Urology	2013	120	6	10.91	1
36	Venous patch graft for Peyronie's disease. Part II: Outcome analysis	El-Sakka, AI	Journal of Urology	1998	120	3	4.62	0
37	Mechanisms of disease: new insights into the cellular and molecular pathology of Peyronie's disease	Gonzalez-Cadavid, NF	Nature Clinical Practice Urology	2005	113	2	5.95	1
38	Ultrastructural-Changes in Impotent Penile Tissue—A Comparison Of 65 Patients	Mersdorf, A	Journal of Urology	1991	113	7	3.42	3
39	Tamoxifen versus placebo in the treatment of Peyronie's disease	Teloken, C	Journal of Urology	1999	112	6	4.48	3
40	Mechanisms of Penile Fibrosis	Gonzalez-Cadavid	Journal of Sexual Medicine	2009	110	2	7.33	7
41	Intratunical Injection of Human Adipose Tissue-derived Stem Cells Prevents Fibrosis and Is Associated with Improved Erectile Function in a Rat Model of Peyronie's Disease	Castiglione, Fabio	European Urology	2013	109	9	9.91	1
42	Potassium paraaminobenzoate (POTAB^TM^)) in the treatment of Peyronie's disease: A prospective, placebo-controlled, randomized study	Weidner, W	European Urology	2005	107	3	5.63	3
43	Epidemiology of Peyronie's disease	Sommer, F	International Journal of Impotence Research	2002	107	7	4.86	0
44	Comparison of gene expression profiles between Peyronie's disease and Dupuytren's contracture	Qian, A	Urology	2004	105	4	5.25	0
45	Evidence based assessment of long-term results of plaque incision and vein grafting for Peyronie's disease	Montorsi, F	Journal of Urology	2000	102	10	4.25	0
46	A First Prospective, Randomized, Double-Blind, Placebo-Controlled Clinical Trial Evaluating Extracorporeal Shock Wave Therapy for the Treatment of Peyronie's Disease	Palmieri, Alessandro	European Urology	2009	101	8	6.73	2
47	The Treatment of Peyronie's Disease with Tamoxifen	Ralph, DJ	British Journal of Urology	1992	101	4	3.16	6
48	Effect of nitric oxide on the differentiation of fibroblasts into myofibroblasts in the Peyronie's fibrotic plaque and in its rat model	Vernet, D	Nitric Oxide-Biology and Chemistry	2002	100	7	4.55	9
49	Phase 2b Study of the Clinical Efficacy and Safety of Collagenase Clostridium Histolyticum in Patients with Peyronie Disease	Gelbard, Martin	Journal of Urology	2012	99	6	8.25	1
50	Peyronie's disease: Prevalence and association with cigarette smoking—A multicenter population-based study in men aged 50–69 years	La Pera, G	European Urology	2001	99	11	4.3	0
51	Psychological Impact of Peyronie's Disease: A Review	Nelson, Christian J.	Journal of Sexual Medicine	2013	98	2	8.91	4
52	Penile traction therapy for treatment of Peyronie's disease: A single-center pilot study	Levine, Laurence A.	Journal of Sexual Medicine	2008	95	3	5.94	4
53	Long-term follow-up of treatment for Peyronie's disease: Modeling the penis over an inflatable penile prosthesis	Wilson, SK	Journal of Urology	2001	95	3	4.13	0
54	Sexual dysfunction in Peyronie's disease: An analysis of 222 patients without previous local plaque therapy	Weidner, W	Journal of Urology	1997	95	4	3.52	0
55	Effects of long-term vardenafil treatment on the development of fibrotic plaques in a rat model of Peyronie's disease	Ferrini, MG	BJU International	2006	94	5	5.22	9
56	Regulation Of the Proliferation and Biosynthetic Activities of Cultured Human Peyronie's Disease Fibroblasts by Interferons-Alpha, Interferons-Beta and Interferons-Gamma	Duncan, MR	Scandinavian Journal of Urology and Nephrology	1991	91	3	2.76	6
57	Combination of Penile Traction, Intralesional Verapamil, and Oral Therapies for Peyronie's Disease	Abern, Michael R.	Journal of Sexual Medicine	2012	89	3	7.42	6
58	Five-Year Follow-Up of Peyronie's Graft Surgery: Outcomes and Patient Satisfaction	Chung, Eric	Journal of Sexual Medicine	2011	89	4	6.85	2
59	Peyronie's disease	Pryor, John	Journal of Sexual Medicine	2004	89	10	4.45	0
60	A rat model of Peyronie's disease associated with a decrease in erectile activity and an increase in inducible nitric oxide synthase protein expression	Bivalacqua, TJ	Journal of Urology	2000	89	8	3.71	3
61	Inflatable Penile Prosthesis Placement in Men with Peyronie's Disease and Drug-resistant Erectile Dysfunction: A Single-Center Study	Levine, Laurence A.	Journal of Sexual Medicine	2010	88	3	6.29	0
62	Use of porcine small intestinal submucosal graft in the surgical management of Peyronie's disease	Knoll, LD	Urology	2001	88	1	3.83	3
63	Is Colchicine Effective in Peyronie's-Disease -A Pilot-Study	Akkus, E	Urology	1994	88	6	2.93	0
64	Peyronie's Disease Following Radical Prostatectomy: Incidence and Predictors	Tal, Raanan	Journal of Sexual Medicine	2010	87	6	6.21	0
65	The use of intralesional clostridial collagenase injection therapy for Peyronie's disease: A prospective, single-center, non-placebo-controlled study	Jordan, Gerald H.	Journal of Sexual Medicine	2008	87	1	5.44	6
66	A surgical algorithm for men with combined Peyronie's disease and erectile dysfunction: Functional and satisfaction outcomes	Mulhall, J	Journal of Sexual Medicine	2005	87	3	4.58	0
67	Use of Penile Extender Device in the Treatment of Penile Curvature as a Result of Peyronie's Disease. Results of a Phase II Prospective Study	Gontero, Paolo	Journal of Sexual Medicine	2009	86	7	5.73	5
68	Surgical correction of Peyronie's disease via Tunica Albuginea plication or partial plaque excision with pericardial graft: Long-term follow up	Taylor, Frederick L.	Journal of Sexual Medicine	2008	86	2	5.38	0
69	Peyronie's disease (CME)	Bella, Anthony J.	Journal of Sexual Medicine	2007	86	4	5.06	0
70	Risk factors for Peyronie's disease: a case-control study	Bjekic, MD	BJU International	2006	86	4	4.78	0
71	Structural alterations in the tunica albuginea of the penis: Impact of Peyronie's disease, ageing and impotence	Akkus, E	British Journal of Urology	1997	85	7	3.15	0
72	Peyronie's disease cell culture models: phenotypic, genotypic and functional analyses	Mulhall, JP	International Journal of Impotence Research	2002	84	4	3.82	0
73	Acetyl-L-carnitine vs. tamoxifen in the oral therapy of Peyronie's disease: a preliminary report	Biagiotti, G	BJU International	2001	84	2	3.65	3
74	Standard Operating Procedures for Peyronie's Disease	Levine, Laurence A.	Journal of Sexual Medicine	2013	83	2	7.55	4
75	Fibrin as an inducer of fibrosis in the tunica albuginea of the rat: a new animal model of Peyronie's disease	Davila, HH	BJU International	2003	83	4	3.95	3
76	Effect of intralesional verapamil for treatment of Peyronie's disease: a randomized single-blind, placebo-controlled study	Shirazi, M.	International Urology and Nephrology	2009	82	5	5.47	0
77	Graft materials in Peyronie's disease surgery: A comprehensive review	Kadioglu, Ates	Journal of Sexual Medicine	2007	82	6	4.82	0
78	Surgical treatment of Peyronie's disease: A critical analysis	Kadioglu, Ates	European Urology	2006	82	6	4.56	3
79	Penile Vascular Evaluation of Men with Peyronie's Disease	Lopez, JA	Journal of Urology	1993	82	2	2.65	0
80	Peyronie's disease	Taylor, Frederick L.	Urologic Clinics of North America	2007	79	2	4.65	0
81	Clinical Safety and Effectiveness of Collagenase Clostridium Histolyticum Injection in Patients with Peyronie's Disease: A Phase 3 Open-Label Study	Levine, Laurence A.	Journal of Sexual Medicine	2015	78	9	8.67	1
82	Comparison of vitamin E and propionyl-L-carnitine, separately or in combination, in patients with early chronic Peyronie's disease: A double-blind, Placebo Controlled, Randomized study	Safarinejad, Mohammad Reza	Journal of Urology	2007	77	3	4.53	0
83	Therapeutic effects of colchicine in the management of Peyronie's disease: a randomized double-blind, placebo-controlled study	Safarinejad, MR	Internatıonal Journal of Impotence Research	2004	77	1	3.85	0
84	Histological and ultrastructural alterations in an animal model of Peyronie's disease	El-Sakka, AI	British Journal of Urology	1998	77	6	2.96	6
85	Treatment of Peyronie's disease with intralesional verapamil injection	Levine, LA	Journal of Urology	1997	76	1	2.81	0
86	Results of surgical treatment for abnormal penile curvature: Peyronie's disease and congenital deviation by modified Nesbit plication (tunical shaving and plication)	Rehman, J	Journal of Urology	1997	75	4	2.78	0
87	Patient-Partner Satisfaction with Semirigid Penile Prostheses for Peyronie's-Disease—A 5-Year Follow-Up-Study	Montorsi, F	Journal of Urology	1993	75	4	2.42	0
88	A New, Innovative, Lengthening Surgical Procedure for Peyronie's Disease by Penile Prosthesis Implantation with Double Dorsal-Ventral Patch Graft: The Sliding Technique	Rolle, Luigi	Journal of Sexual Medicine	2012	74	8	6.17	5
89	The prevalence of Peyronie's disease in diabetic patients with erectile dysfunction	Arafa, M	International Journal of Impotence Research	2007	74	5	4.35	0
90	The results of plaque incision and venous grafting (Lue procedure) to correct the penile deformity of Peyronie's disease	Kalsi, J	BJU International	2005	74	4	3.89	0
91	Clinical presentations of Peyronie's disease	Pryor, JP	International Journal of Impotence Research	2002	74	2	3.36	0
92	Application of pericardial graft in the surgical management of Peyronie's disease	Hellstrom, WJG	Journal of Urology	2000	74	2	3.08	0
93	Venogenic Impotence Following Dermal Graft Repair for Peyronie's Disease	Dalkin, BL	Journal of Urology	1991	74	2	2.24	0
94	Surgery for Peyronie's disease	Levine, Laurence A	Asian Journal of Andrology	2013	73	2	6.64	0
95	Verapamil versus saline in electromotive drug administration for Peyronie's disease: A double-blind, placebo-controlled trial	Greenfield, Jason M	Journal of Urology	2007	73	3	4.29	3
96	A critical analysis of nonsurgical treatment of Peyronie's disease	Hauck, Ekkehard W.	European Urology	2006	72	4	4	6
97	A case-control study on risk factors for Peyronie's disease	Carrieri, MP	Journal Of Clinical Epidemiology	1998	72	5	2.77	3
98	Simultaneous Penile Lengthening and Penile Prosthesis Implantation in Patients with Peyronie's Disease, Refractory Erectile Dysfunction, and Severe Penile Shortening	Sansalone, Salvatore	Journal of Sexual Medicine	2012	71	8	5.92	5
99	Expanding the paradigm for plaque development in Peyronie's disease	Mulhall, JP	International Journal of Impotence Research	2003	69	1	3.29	0
100	Restoration of Penile Function and Patient Satisfaction with Intralesional Collagenase Clostridium Histolyticum Injection for Peyronie's Disease	Ziegelmann, Matthew J.	Journal of Urology	2016	68	6	8.5	2

PN, Publication Name; CA, Corresponding Author; ST, source title; PY, publication year; TC, total citations; NoA, number of authors; ACI, Adjusted Citation Index; AmS: Altmetric score.

**Figure 1:**
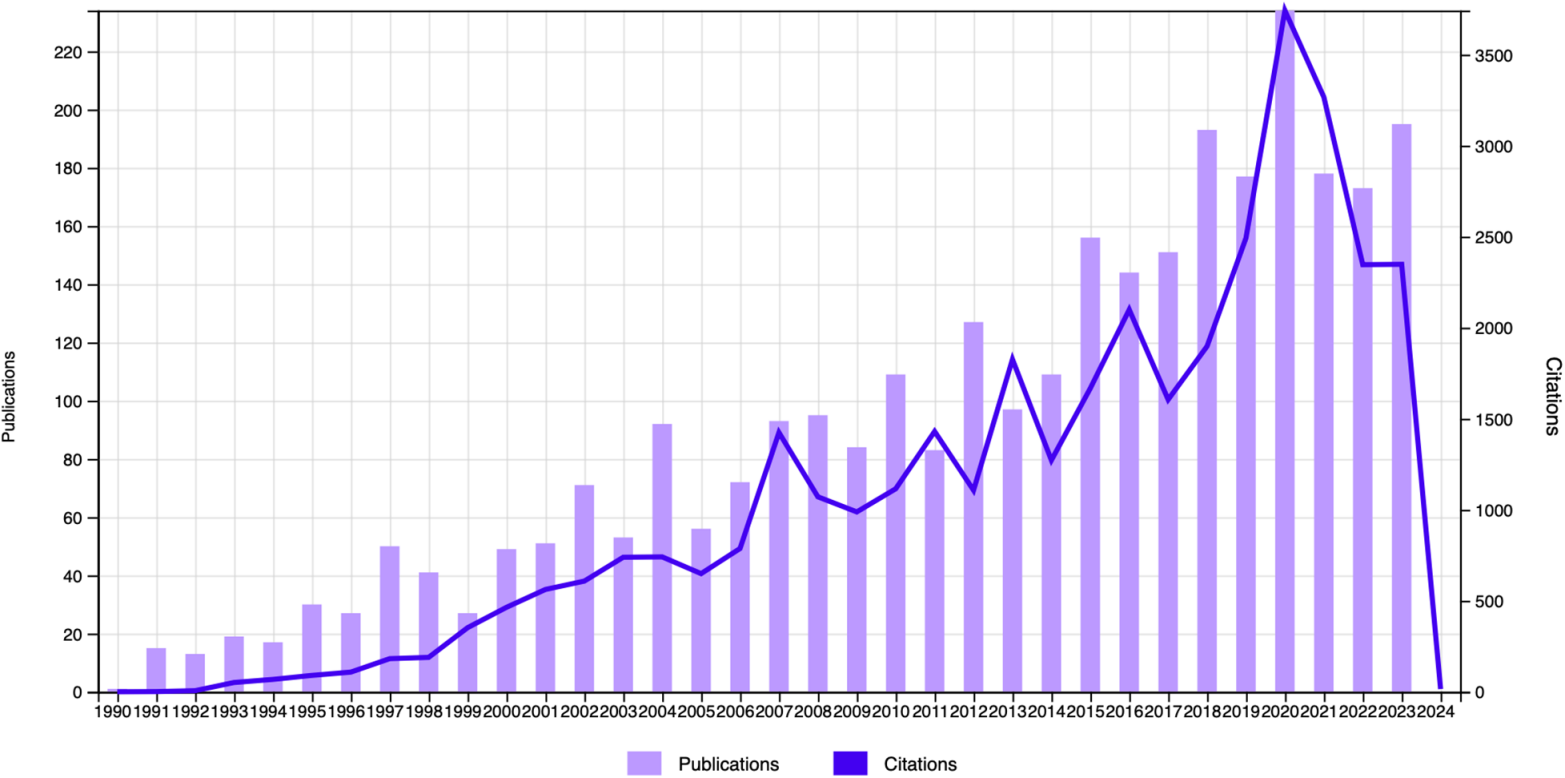
The total number of citations and articles by year.

**Figure 2:**
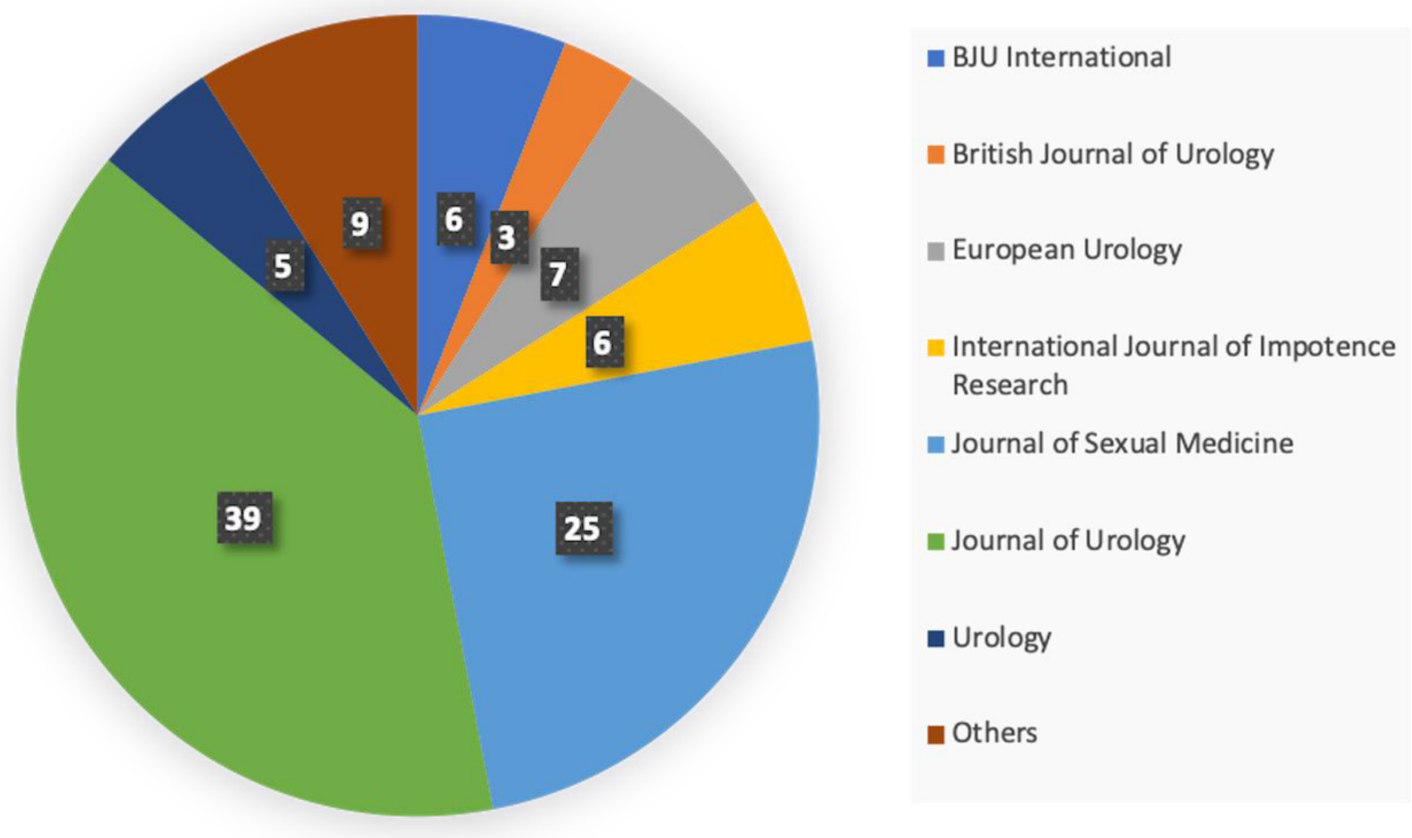
Journal distribution of the T100 articles (number and % out of 100).

The highest number of papers were published in 2020, according to an analysis of the articles' date of publication, and the maximum number of citations were also found in 2020. (Figure 1). When the journals with the highest portion of the T100 articles were examined, the *Journal of Urology* was found to have the most publications, followed by the *Journal of Sexual Medicine* (Figure 2).

**Figure 3:**
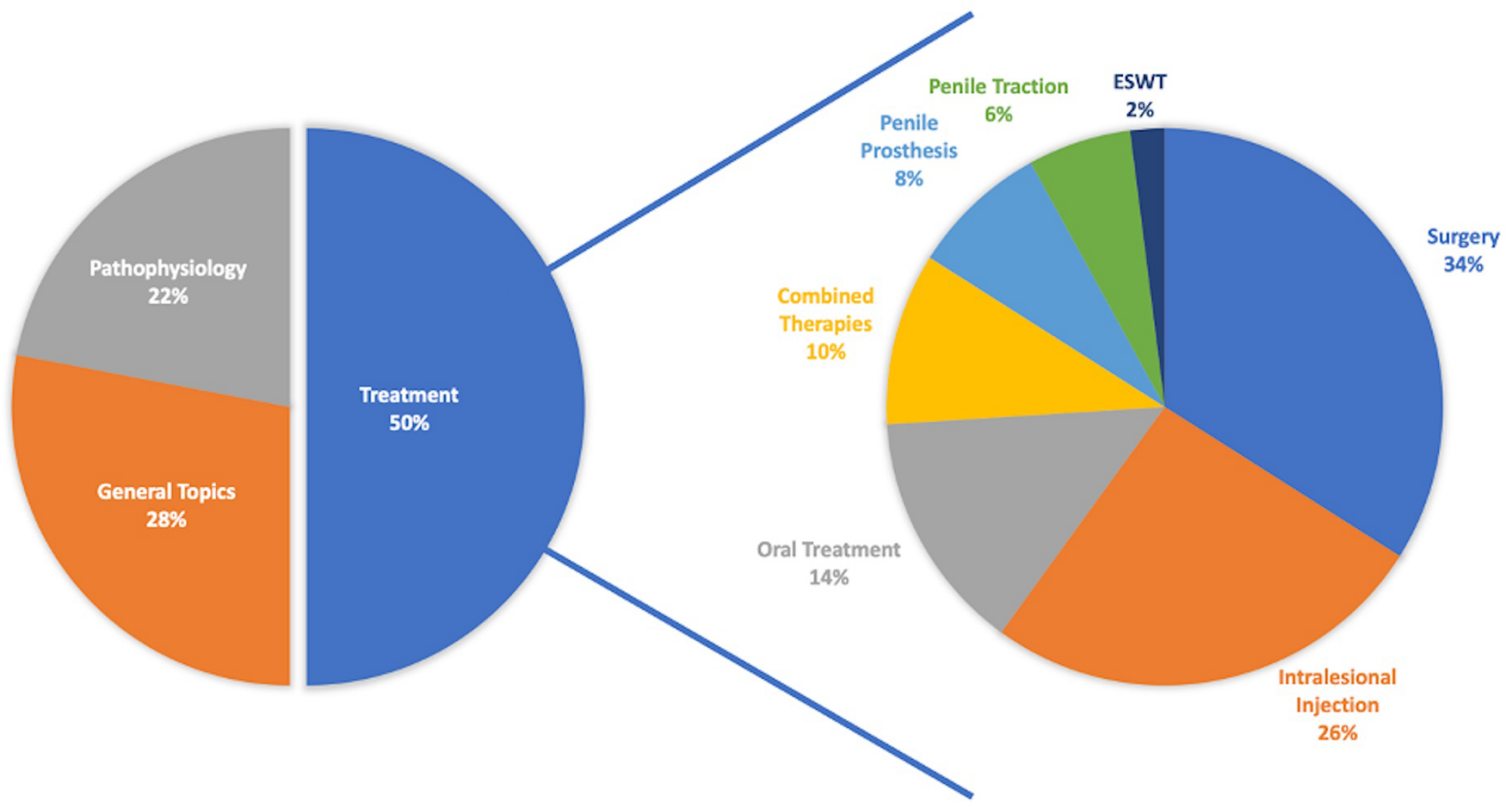
Journal distribution of the T100 articles according to the topic.

One-half of the T100 journals focused on the treatment of PD, while 28% focused on general topics such as epidemiology, prevalence, and recommendations, and 22% examined pathophysiology. Concerning treatment, 34% of the T100 articles examined surgery, 26% concentrated on intralesional injections, and 14% focused on oral treatments (Figure 3). The studies included in the T100 were mainly concerned with recommendations, prevalence, relationship with trauma, intracavernous injections, and etiology.

**Figure 4:**
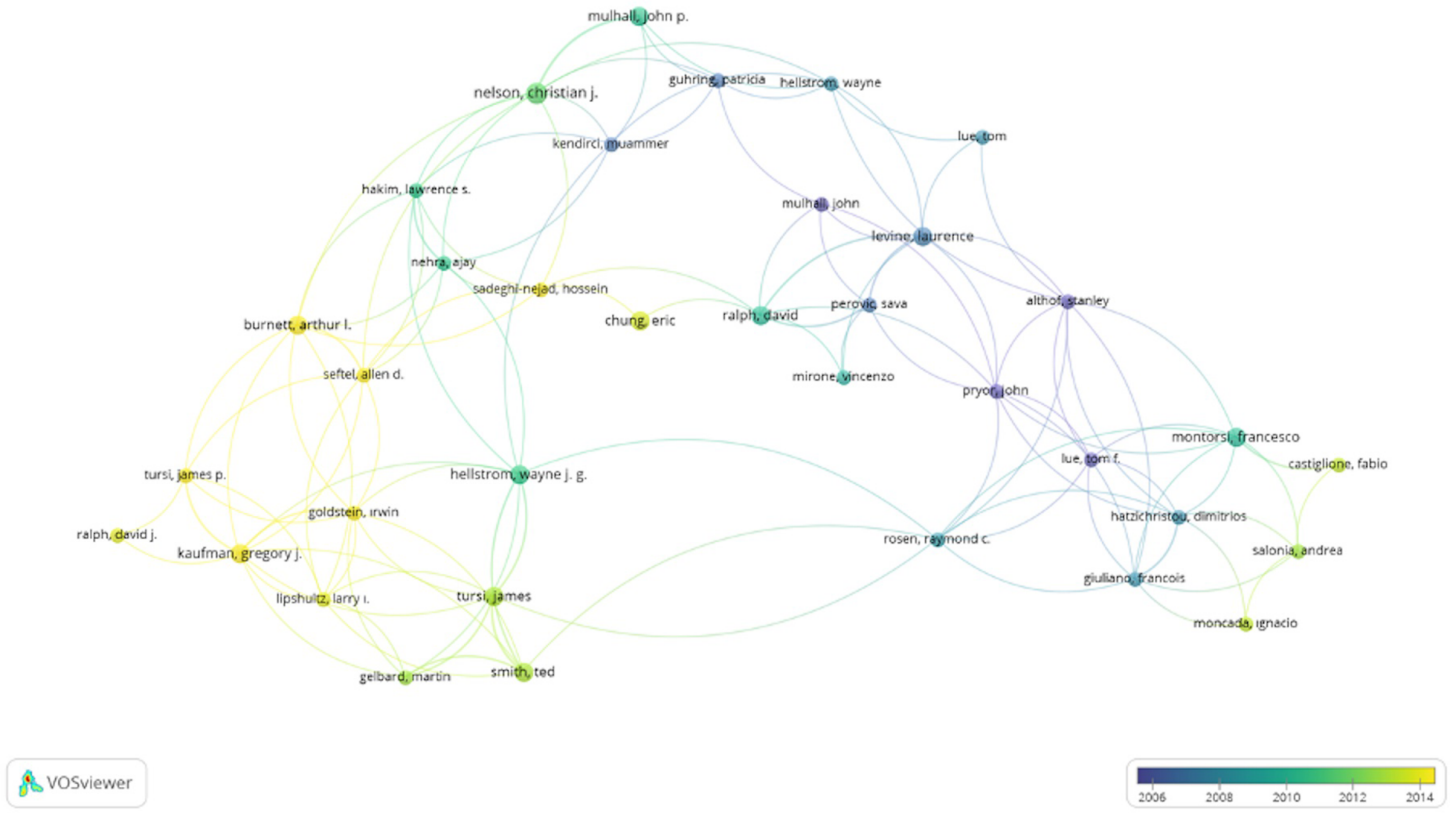
Co-authorship analysis for the authors (minimum number of articles: 2 and above, and minimum number of citations of an author: 2 and above).

**Figure 5:**
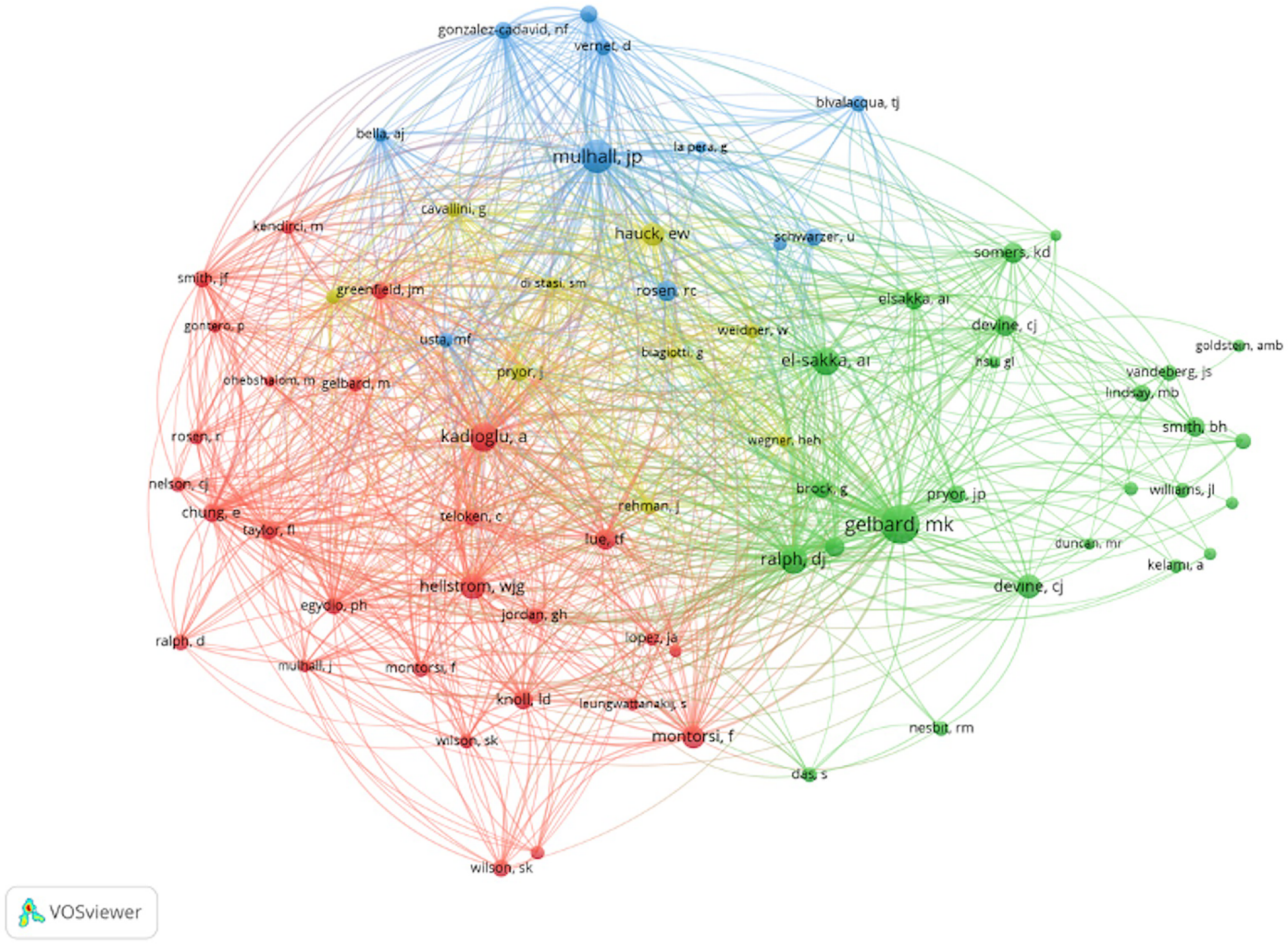
Co-citation analysis for the authors (minimum number of citations: 10 and above).

Analysis of the authors of the T100 articles showed that the author with the highest total number of citations was Lue TF, MD, with a total of 1,357 citations and 11 articles, while the author with the highest number of articles was Levine Laurence A, MD, with 9 articles. Co-authorship and co-citation analysis for the authors is presented in Figure 4 (minimum number of articles: 2 and above) and Figure 5 (minimum number of citations: 10 and above).

**Figure 6:**
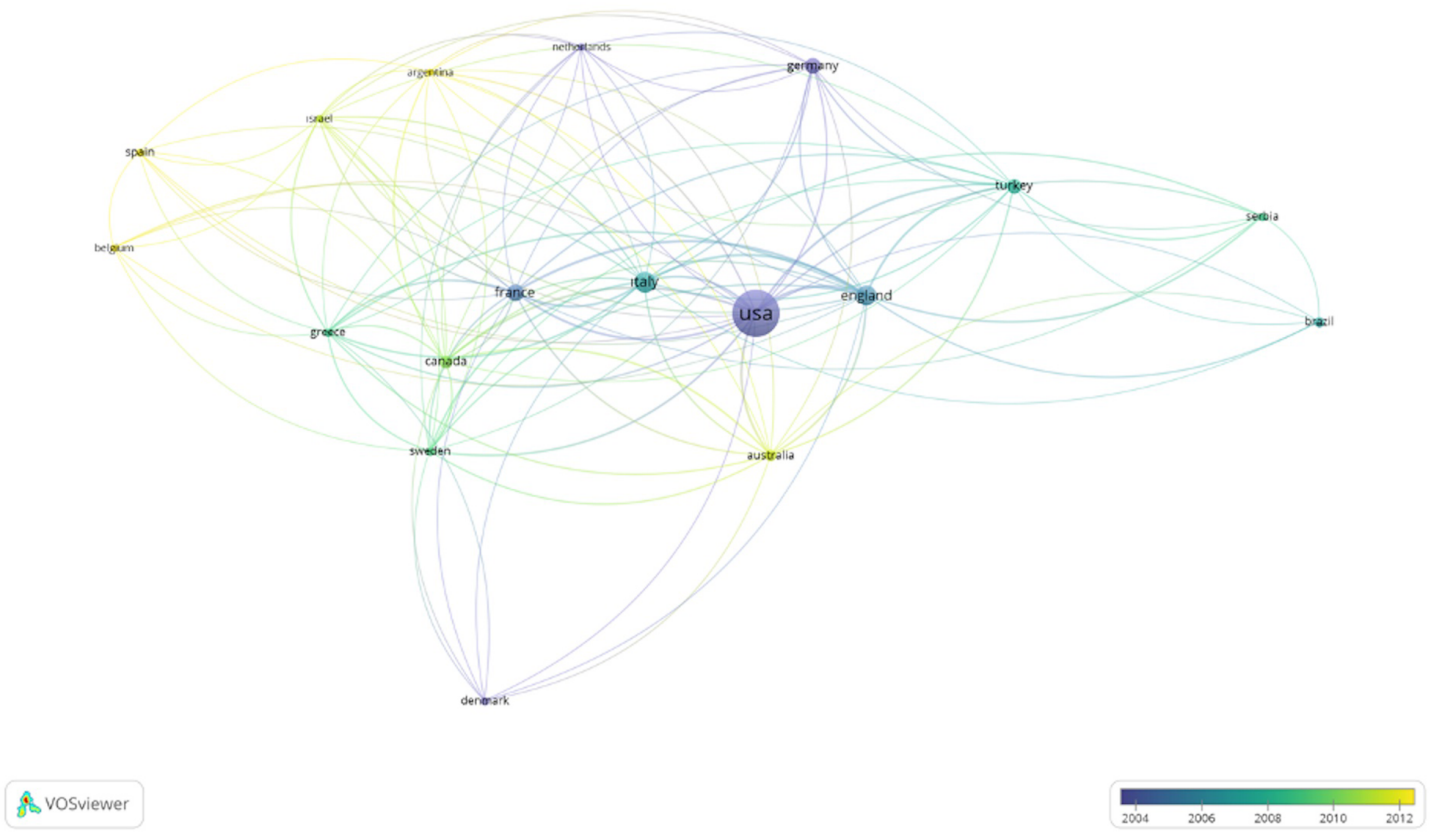
Co-authorship analysis for the authors according to country (minimum number of articles of a country: 2 and above).

As expected, all of the articles were published in English. The distributions of articles according to country was as follows: 64% US, 11% Italy, and 25% others (Germany, England, Turkey, etc.) (Figure 6).

**Figure 7:**
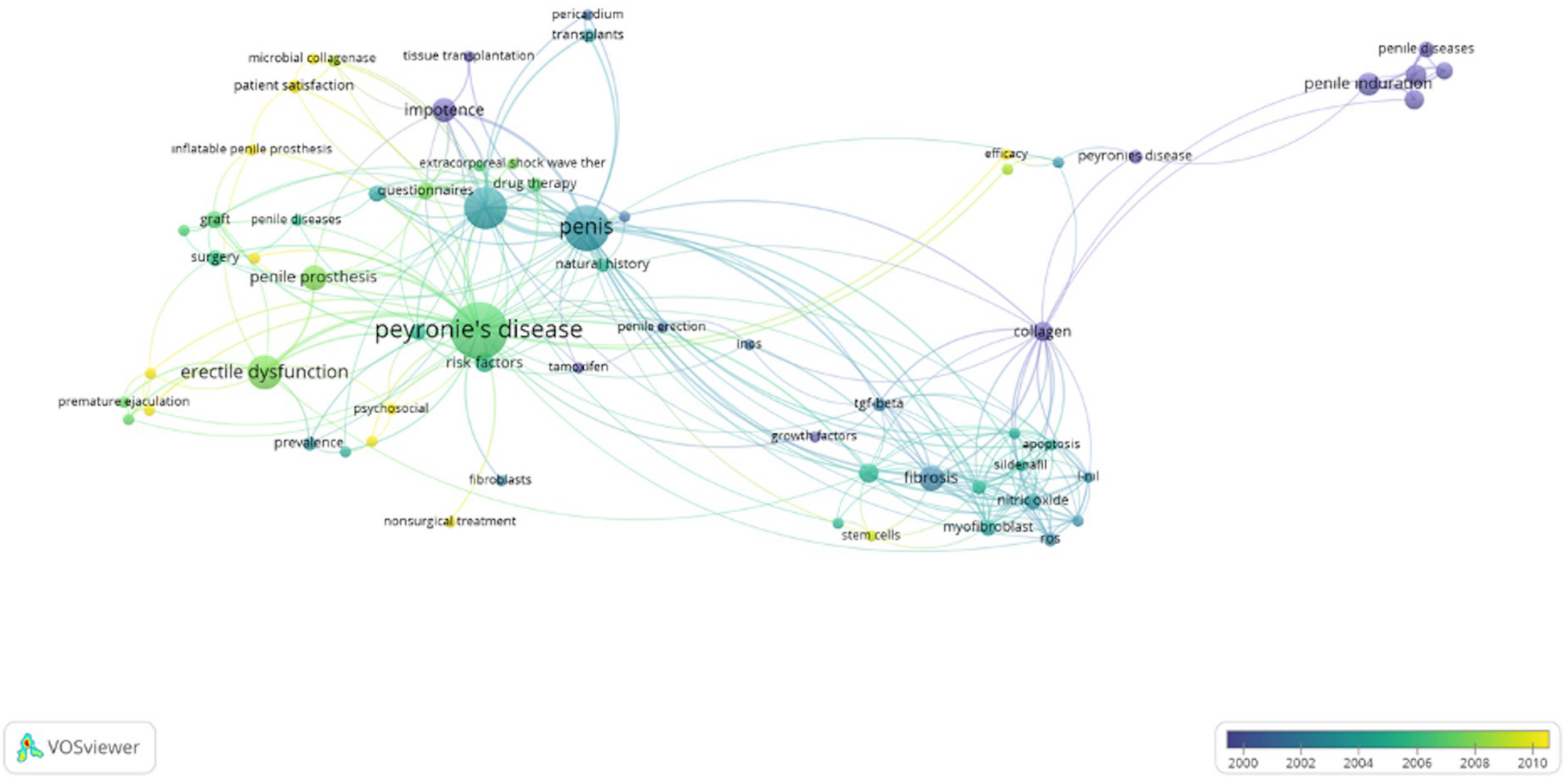
The analysis of the top keywords in a density format (minimum number of author occurrences of a keyword: 2 and above).

The analysis of keywords showed that the top 10 keywords were as follows: Peyronie's disease, penis, penile induration, erectile dysfunction, fibrosis, penile prosthesis, impotence, collagen, tunica albuginea, and risk factors (Figure 7).

## Discussion

Bibliometric analysis is useful for several reasons. For example, it can identify and highlight the contributions of colleagues and forebearers, acknowledge significant urological advancements, and provide a helpful historical context for current trends in the field. The urological literature can be examined using citation analysis, which also reveals quantitative data about authors, subjects, countries, and journals that are useful for finding classic works and high-impact publications (6).

Altmetric analysis is a method that tracks the influence of a publication in the online area, rather than within academic circles. It enables us to quantify the extent to which a scientific study is referenced across different online platforms, including social media, blogs, news outlets, and podcasts. Additionally, it measures the number of online views and downloads. Most of the publications about PD' AmS are found to be very low. But in our study, like Kolahi et al., we found a positive but weak correlation between the number of citations and AmS (7). However, it is important to acknowledge that altmetrics is a relatively recent notion within the academic community. Furthermore, the growing awareness and use of social media among researchers in the future may potentially amplify this association. This shows that this issue is a phenomenon that has gained a place in the academic world rather than being a popular problem among people.

Many factors influence the number of citations an article receives. Articles typically receive the most citations 3–10 years after their publication (8). Older articles are more likely to be cited (9), but when detailed analyses were performed in the current study, it was seen that most articles and citations both occurred in 2020. As such, this year can be considered to be a milestone in PD research, generating a better understanding of the disease, including its etiology, anatomy, predisposing factors, histological structure, and surgical and non-surgical techniques. None of the most cited articles were published in the last five years. More than one-half of the publications in the T100 were published in the *Journal of Urology* and *Journal of Sexual Medicine*, both of which have a high impact factor and h-index. This could be a sign that scientists want to publish their works in prestigious publications related to PD. These two journals have a considerably great impact on studies that examine PD and can be regarded as references for articles listed in the T100. Most of the studies were conducted by individual scientists, and a few studies in the T100 were found by a study group using a multicenter database. Contrary to collaborative disciplines such as urooncology or urogynecology, multicenter studies on andrology and PD are needed rather than single-center studies.

We also found that one-half of the papers on PD concerned treatment, while older publications mostly focused on pathophysiology. Newer studies focused on new surgical methods and approaches. Among these studies, surgeries were the most common subject, followed by intralesional injections. The publication with the highest number of citations was also included in this section. Intralesional CCH has been used in the treatment of PD since 1985, with the author who published the first article being the same author of the most cited article (10). In a randomized controlled trial, Gelbard et al. stated that intralesional CCH provided a significant improvement in plaque size and penile deformity compared to a placebo (11). This article also occupied the 15th place in the T100 list. Intralesional verapamil (12) and interferon alpha-2b (13) are the other treatment modalities seen in the T100 list with the most citations. Although the most cited publication was on intralesional injections, the most cited articles on treatment options concentrated on surgical methods. The Nesbit technique is the oldest surgical method used in the treatment of PD (14). However, in the publications on the T100 list, articles about graft materials stood out as a new trend. When examined in a more limited group, we can see that the first 10 articles were generally related to etiology and pathophysiology. For this, it was observed that there are very few surgeries and interventional procedures, and only one publication was found to be about injections. Since most of these subjects have been examined in relation to PD, multicenter randomized prospective studies about surgery are needed.

The US had the highest number of articles in the T100. This is not surprising since the studies with the greatest funding are from the US (15). According to a review of co-authorship analysis for authors according to country using VOSviewer, the US had the highest link strength. Christiansen et al. found that only 15.1% of articles written about PD had a funding source in 2020. Other articles appeared to be self-funded (15). This may suggest that it is easier to perform research in high-income countries, such as the US and some European countries, compared to other, developing countries. A newly published article about Peyronie's disease and bibliometric analysis has been published, but it has not been focused on funding issues, which we think are very important for scientific developments (16). A total of three studies from our country, Turkey, are included in this list, and although surgery is frequently performed in Turkey, they are in the category of current literature analysis or review articles due to lower income and difficulties in drug supply. We believe that further studies will be interrupted and new developments will be stymied due to a lack of funding on such a developmental subject in andrology. In our study, 19% of the T100 studies had a funding source that slightly increased in two years. This finding leads us to the conclusion that good material resources facilitate the development of science, and that this increase in funding provides hope for the future of research on PD. By doing more research on the pathological sequence of cellular and molecular processes and deepening our comprehension of the pathophysiology of PD via animal models, the achievement of new and efficient medical treatments and management modalities will become a feasible goal.

This study had some limitations. First, the study eliminated other databases like Google Scholar and Scopus, which would have had an impact on the quantity of papers returned, and we only used the Web of Science database. Second, self-citations and interactions between citations were not examined. Third, we only investigated the top 100 articles with the most citations, so it is possible that we missed some more significant articles with fewer citations.

## Conclusion

Our study was the first to analyze the top 100 most cited studies about Peyronie's disease (PD). In the T100 list, the *Journal of Urology* and the *Journal of Sexual Medicine* were the leading journals. The vast majority of the articles were focused on treatments of the disease, with surgery being the predominant topic. The US was the leading country with the most publications. Future research is anticipated to benefit from and be guided by the insights produced in this study. Novel therapies like stem cells, new greft materials with new plication techniques, and new medical treatment modalities are interesting hot research topics. Lastly, there is a need for further studies that include larger databases and receive greater funding.

## Data Availability

The original contributions presented in the study are included in the article/Supplementary Material, further inquiries can be directed to the corresponding author.

## Author's note

In this article, MFŞ and ÇD get part at substantial contributions to conception and design, MA and SŞ at drafting and revising the article critically for important intellectual content and CMY and HSD get part at final approval of the version to be published.
